# A report from the Second Tanzania Liver Cancer Conference (TLCC) 2025 – leading the fight against liver cancer in Sub-Saharan Africa

**DOI:** 10.3332/ecancer.2026.2067

**Published:** 2026-01-22

**Authors:** Ally H Mwanga, Jeanine Justiniano, Eric M Mbuguje, Balowa Musa, Deogratius B Mwanakulya, Andrew Swallow, Edith Kimambo, Eva Uiso, Swaleh Pazi, Latifa Rajab, Nashivai E Kivuyo, Larry Akoko, Azza Naif, Advera Ngaiza, Sara Nyagabona, Jerry Ndumbalo, Amos R Mwakigonja, Jim E Littlejohn, Seif Wibonela, Cameron E Gaskill

**Affiliations:** 1 Department of Surgery, Muhimbili University of Health and Allied Sciences , PO BOX 65001, Dar es Salaam, Tanzania; 2 Department of Surgery, Muhimbili National Hospital, PO BOX 65000, Dar es Salaam, Tanzania; 3 Department of Surgery, University of California Medical Center, 4301 X St, Sacramento, CA 95817, USA; 4 UC Davis Center for Global Surgery, 2335 Stockton Blvd, 6th Floor, Sacramento, CA 95817, USA; 5 Department of Interventional Radiology, Muhimbili National Hospital, PO BOX 65000, Dar es Salaam, Tanzania; 6 Division of Oncology, Department of Internal Medicine, Muhimbili National Hospital, Post box 65000, Dar es Salaam, Tanzania; 7 Division of Gastroenterology, Department of Internal Medicine, Muhimbili National Hospital, PO BOX 65000, Dar es Salaam, Tanzania; 8 Department of Interventional Radiology, Ocean Road Cancer Institute, Ocean Road, PO BOX 3592, Dar es Salaam, Tanzania; 9 Division of Pathology, Department of Internal Medicine, Muhimbili National Hospital, PO BOX 65000, Dar es Salaam, Tanzania; 10 Department of Oncology, Ocean Road Cancer Institute, Ocean Road, PO BOX 3592, Dar es Salaam, Tanzania; 11 Department of Anesthesiology, University of California Medical Center, 4301 X St, Sacramento, CA 95817, USA; 12 Surgical Skills Laboratory, Muhimbili University of Health and Allied Sciences, PO BOX 65001, Dar es Salaam, Tanzania

**Keywords:** liver cancer, Tanzania, global cancer, surgical oncology, hepatocellular carcinoma

## Abstract

The Second Tanzania Liver Cancer Conference (TLCC) took place on 25–26 July 2025 in Dar es Salaam, Tanzania with the aim of uniting healthcare providers to continue the fight against liver cancer in Tanzania. The conference focused on the following agenda items: 1) To build awareness among local and international healthcare providers on the status of liver cancer in Tanzania, and the diagnostic and management options afforded to them. 2) To promote a multidisciplinary approach to the clinical care of patients with liver cancer in Tanzania. 3) To foster local and international collaboration in liver cancer research and care. TLCC 2025 was preceded by community-facing pre-conference activities, including hepatitis B virus screening and vaccination, research network symposium and 2 day surgical camp. The conference was attended by 247 healthcare professionals from varying disciplines, featuring 38 local and international speakers who comprehensively covered a wide range of topics related to research and clinical care of liver cancer patients. Overall, the conference was well-received by attendees. As Tanzania's second conference on the subject, TLCC 2025 marked continuing efforts to lead the fight against liver cancer in Sub-Saharan Africa.

## Introduction

Building on the tremendous success of the inaugural Tanzania Liver Cancer Conference (TLCC) in 2023 [1] – which welcomed 160 attendees and achieved a 100% satisfaction rate among respondents – the Tanzania Liver Cancer Group (TLCG) hosted the second

**Figure 1:**
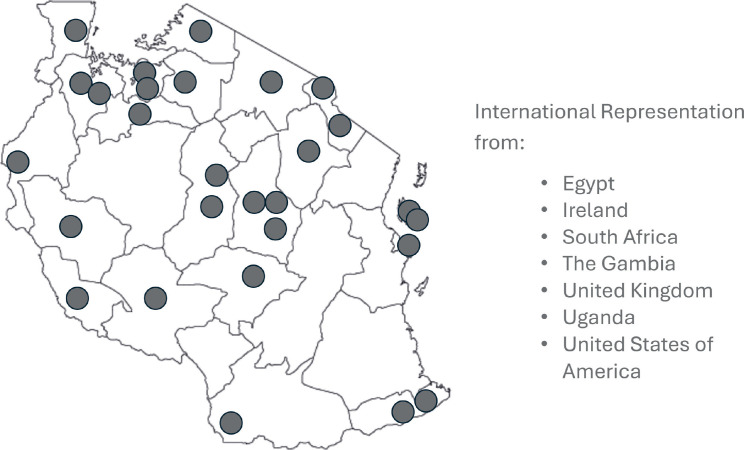
Representation of attendees/speakers at TLCC 2025

TLCC on 25–26 July 2025 in Dar es Salaam, Tanzania. TLCG is a multi-institutional team of surgeons, interventional radiologists, diagnostic radiologists, pathologists, gastroenterologists and medical oncologists that formed a working group in 2022 to address liver cancer in Tanzania through a multidisciplinary approach. The goal of the conference was to unite in the fight against liver cancer. There were 247 recorded attendees at the conference from various disciplines across Tanzania and abroad (Figure 1). The conference featured 38 speakers from Tanzania, Egypt, The Gambia, South Africa, Ireland, Uganda and the United States (USA). It provided a comprehensive overview of the state of liver cancer in Tanzania.

Since the first TLCC in 2023, the TLCG has established a multidisciplinary liver tumour board (MLTB) at Tanzania’s largest hospital and national referral center, Muhimbili National Hospital (MNH). The MLTB has evaluated almost 300 patients with liver cancer across Tanzania in the span of 2 years. Despite these promising developments, the overall awareness of the diagnostic and treatment options available to liver cancer patients from the national perspective remain severely limited. The TLCC 2025 was dedicated to ‘Leading the Fight against Liver Cancer in sub-Saharan Africa (SSA)’. The overall themes of the conference were as follows:

1. To build awareness among local and international healthcare providers on the status of liver cancer in Tanzania, and the diagnostic and management options afforded to them.
2. To promote a multidisciplinary approach to the clinical care of patients with liver cancer in Tanzania.
3. To foster local and international collaboration in liver cancer research and care.

## Background of liver cancer in Tanzania

Liver cancer is the third leading cause of cancer-related mortality worldwide, with over 866,000 cases, nearly 80% of which occur in low- and middle-income countries (LMICs) [2]. Primary liver cancers include hepatocellular carcinoma (HCC) (75%–80% of cases), cholangiocarcinoma (10%–15%) and other rare pathologies (e.g., angiosarcoma and fibrolamellar carcinoma) [3]. While other primary liver cancer rates are likely underreported, HCC accounts for the large majority of the liver cancer in Africa with pathogenesis predominantly driven by viral Hepatitis B (HBV) and Hepatitis C, aflatoxin exposure and alcohol consumption [4, 5]. The global burden of HCC falls disproportionately on resource-limited settings, with 80% of cases occurring in LMICs, particularly in SSA and East/Southeast Asia [6]. Furthermore, HCC tends to manifest at a younger age in Africa, with a median age of onset at 45 and affecting individuals as young as 30, producing large loss of Disability Adjusted Life Years and significant burden on an already under-resourced healthcare system [7]. In SSA, an estimated 46,000 new liver cancers occur each year [8]. Liver cancer is the third most common cause of cancer death in Tanzania [9]. While the exact incidence is unknown, a report of 142 HCC cases from Bugando Hospital in Tanzania, published in 2014, demonstrated that most HCC cases presented at a late stage and no patients received curative therapy [10].

## Pre-conference activities

### Hepatitis B virus screening and vaccination

In a recent study published in The Lancet, the Commission addressed the global HCC burden and recommended different strategies on the prevention and treatment of this disease, stating that ‘60% of liver cancers are preventable via modifiable risk factors, including HBV’ among many others [11]. They recommend universal vaccination for HBV and screening measures to reduce HCC burden globally. Dr. Eva Uiso (Hepatology, Tanzania), with a team of residents, nurses and laboratory technicians, provided HBV screening for a total of 260 clients. Two clients tested positive for HBV infection and were referred to the MNH Hepatology Clinic for further evaluation and follow up. The 258 that screened negative for HBV received the first dose of HBV vaccine. In addition, 111 clinical medical students were vaccinated with the third dose of HBV as they all had previously received 1st and 2nd doses. Therefore, the total number of vaccinations done in one day of preconference activities for the TLCC 2025 was 369.

### Research network symposium

Dr. Erick Mbuguje (Interventional Radiology (IR), Tanzania), Prof. Ponsiano Ocama (Hepatology, Uganda), Dr. Fidel Rubagumya (Oncology, Rwanda), Dr. Nimzing Ladep (Hepatology, United Kingdom), Dr. Julius Mwaiselage (Oncology, Tanzania), Dr. Jerry Ndumbalo (Clinical and Radiation Oncologist, Tanzania), Prof. Amos Mwakigonja (Pathology, Tanzania) and Dr Sima Rugarabamu led a day dedicated to a Research Network Symposium at MNH with the more than 180 attendees from all specialties at the hospital with the purpose of establishing a network to support HCC research in SSA. The question of the day was: ‘Can Africa Unite in the Fight Against Liver Cancer’. The goal of the symposium was to facilitate collaboration, strengthen partnerships, share knowledge, identify research priorities, promote capacity building, develop actionable outcomes and enhance awareness. The day included round table discussions on the goals and obstacles in liver cancer research in Tanzania, expanding multidisciplinary care, establishing a registry and finally, collaborating across Africa and beyond. Actioned plans were to establish association on hepatobiliary disease in East Africa, collecting same data information on existing multidisciplinary tumour board with the aim of forming East Africa Multidisciplinary tumour board and Hepatobiliary tumour registry, Collaborative research activities in East Africa as they have similar geographical characteristic starting with disease demographic data.

### Surgical mentoring

Dr. Ally Mwanga (Surgical Gastroenterology, Tanzania), Dr. Andrew Swallow (Surgical Gastroenterology, Tanzania), Dr. Hossam Soliman (Hepatobiliary Surgery, Egypt), Dr. Islam Ayoub (Hepatobiliary Surgery, Egypt) and Dr. Cameron Gaskill (Surgical Oncology, USA) held a 2-day surgery camp where they provided surgical care for 4 patients. Surgeries included a left hemihepatectomy for intrahepatic cholangiocarcinoma, an extended right hemihepatectomy for a pediatric patient, a left hemihepatectomy in a cirrhotic patient with HCC and a pancreaticoduodenectomy (Whipple) operation for pancreatic cancer. All operations were attended by and assisted by Muhimbili University of Health and Allied Sciences (MUHAS) surgical faculty and residents.

## Conference

### Opening ceremony

The Chairman of the TLCG, Dr. Ally Mwanga (Surgical Gastroenterology, Tanzania) outlined the group’s efforts in a presentation titled ‘Multidisciplinary, Cross-Institutional Collaboration’. He discussed the beginning of the TLCG brought on by fragmented care, lack of data, shortage of liver cancer research in Tanzania and the need for community awareness. He also emphasised the goals of the conference: Implement a national MLTB, create national liver cancer guidelines, improve research efforts and strengthen collaborations. This was followed by motivational words from the guest of honor, Dr. Diwani Msemo (Oncology and Palliative Care, Tanzania) as a representative of the Minister of Health Hon. Jenista Mhagama. He emphasised the Ministry of Health’s ongoing fight against liver cancer, notably through the Hepatitis B Vaccination program (since 2002). In a keynote address, the ambassador of the United Republic of Tanzania in the United States Dr. Elsie S. Kanza, sent her regards and emphasised the importance of raising awareness on liver cancer in Tanzania, highlighting the importance of our noble work and continual efforts. Finally, Dr. Larry Akoko (Surgical Oncology, Tanzania) addressed the conference attendees and reported on the epidemiology of liver cancer in Tanzania and SSA, and highlighted prevention and control strategies for HCC in Africa.

### Session: liver cancer in Dar es Salaam, Tanzania

This session highlighted the ongoing clinical and research efforts of TLCG members, primarily working at MNH. Discussion began on the current state of screening and diagnosis, IR, surgery and transplant, medical oncology and pathology in Tanzania by Dr. Lufti Abdallah (MNH, Tanzania), Dr. Eric Mbuguje (MNH, Tanzania), Dr. Andrew Swallow (MNH, Tanzania), Dr. Edith Kimambo (MNH, Tanzania) and Dr. Amos Mwakigonja (MNH, Tanzania), respectively. Dr. Abdallah discussed screening efforts around rural areas of Tanzania. He also emphasised the limitations in diagnostics around Tanzania, with alpha-feto protein laboratory and certain imaging modalities such as CT scanners and MRIs not being available in certain parts of the country. Dr. Mbuguje’s session highlighted the current status of IR in Tanzania and the management of liver cancer. He discussed the current IR therapies still unavailable in Tanzania, such as ablation and y90 embolisation. Dr. Swallow discussed the advancements of liver resection at MNH, efforts to begin liver transplantation in the country. Dr. Kimambo provided an overview of oncology at MNH and the available chemotherapies, immunotherapies and molecular-targeted therapies offered, such as sorafenib, lenvatinib, pembrolizumab, bevacizumab, FOLFOX4 and doxorubicin. Unfortunately, newer drugs approved for the treatment of HCC are not available and are very expensive. Thereafter, Dr. Mwakigonja concluded this session by providing an overview of the pathology of HCC at MNH and future opportunities in the field of pathology, such as digital pathology and the use of artificial intelligence.

### Session: liver cancer around Tanzania

This program featured clinicians from major medical centers across Tanzania, highlighting treatment capacity and outcomes of each region. Dr. Felician Kachinde (Lake Zone, Bugando Medical Center) shared the experience of developing the surgical capacity to perform liver resections at BMC, reporting that the first major liver surgery, a hemihepatectomy, was performed May 2024. Dr. Samwel Wambura (Central Zone, Benjamin Mkapa Hospital) presented on the current status of liver cancer in Dodoma. Dr. Wambura spoke about the beginning of their tumour board, their liver cancer registry and emphasised the availability of radiotherapy at their institution starting next year. Dr. Mathias Banzi (Southern Highlands, Mbeya Zonal Referral Hospital) presented on the management of liver cancer in the Southern Highlands. Dr. Banzi reported a total of 80 patients with primary liver cancer treated between January 2023 to June 2025 at their facility, primarily HCC. He also mentioned that capecitabine is the leading drug used for liver cancer patients at MZRH due to financial toxicity among the population.

### Session: liver cancer in Africa

In this session, speakers from around SSA were invited to share their country’s experience with HCC and the ongoing programmatic efforts to address the growing disease burden. A special emphasis was placed on learned lessons to shared problems with the goal of cross collaboration within SSA in fighting HCC. Dr Lamin Jaiteh (Surgical Oncology, the Gambia), presented on the epidemiology of HCC in the Gambia, affecting young men disproportionately. Dr. Jaiteh discussed the hardships of treating HCC in the Gambia, with patients presenting as Barcelona Clinic Liver Criteria (BCLC) >B. He mentioned that current treatment modalities still not available, such as transarterial chemoembolization (TACE) and transplant. Finally, Dr. Jaiteh highlighted his country’s efforts to strengthen prevention through HBV immunisation, introducing surveillance programs and expanding treatment access. This presentation was followed by Dr Edward Jonas (Surgical Gastroenterology, South Africa), who discussed building HCC treatment capacity in South Africa. Dr. Jonas reported the current lack of systemic therapy in South Africa, highlighting a problem treating the majority of liver cancer patients in the country who present as BCLC >B. He further reported on efforts to expand TACE eligibility outside of the BCLC guidelines to make up for this resource constraint and emphasised the need to re-evaluate the applicability of BCLC in SSA. Finally, Dr. Hamdy Azim (Oncology, Egypt), presented on the challenges and opportunities in the management of HCC in 2025. He reviewed a patient case who presented to his clinic with HCC and delineated the available treatment modalities in Egypt, specifically the targeted molecular therapies used for treatment.

### Session: Fighting HCC in Tanzania

This session focused on efforts to improve the treatment capacity of HCC in Tanzania. Dr. Balowa Musa Baraka (IR, Tanzania) presented on IR in The Non-Western World. He presented the beginnings of IR in Tanzania back in 2017 through Road2IR consortium. Dr. Jeanine Justiniano (General Surgery Resident, USA) presented on the inception of a liver surgery curriculum composed of didactics and a bovine liver simulation lab. Dr. Justiniano demonstrated the curriculum as an inexpensive, sustainable and effective model for liver surgery simulation in low resource settings. Dr. Anette Joseph Kessy (Radiology, Tanzania) presented on the role of ultrasound in early diagnosis of HCC and emphasised the benefits of ultrasound including being inexpensive, widely available, real-time assessment, non-invasive, non-radiating and easily portable. Dr. Nashivai Kivuyo (General Surgeon, Tanzania) presented on the partnerships between LMICs and HICs in capacity building efforts and research collaboration, with a shared goal of global health equity for all. Dr. Cameron Gaskill (Surgical Oncology, USA) presented the results of the national stakeholder survey on tumour boards throughout Tanzania and a nationwide, comprehensive liver cancer capacity assessment. This presentation highlighted the current status of liver cancer management in Tanzania, and emphasised areas of improvement to better care for liver cancer patients in the country.

### Session: the impact of a MLTB

As a main activity of the TLCG, the Tanzania Liver Tumour Board activities were Jennifer Dent, BSc, MBA (BIO Ventures for Global Health, USA) presented on the Impact of Implementing a Tumour Board. She discussed its role in improving treatment planning, supporting physician decision making and promoting collaboration. She emphasised the positive impacts on patient outcomes in Africa. Dr. Ferdinand Matemu (Surgical Gastroenterology Fellow, Tanzania) proceeded with a mock tumour board case discussion of a 66 year old female patient presenting to MNH with a liver tumour, diagnosed with cholangiocarcinoma. Multidisciplinary discussion was held to review the case and determine consensus recommendations.

### Session: bridging to high-income countries

In this session, international experts were invited to share their country’s experience with HCC care, with an emphasis on providing insight into capacity and program development to aid strategic planning for HCC care in Tanzania. Dr. Martin Corbally (Royal College of Surgeons in Ireland, Ireland) presented on surgical considerations for hepatoblastoma in children. Dr. Cameron Gaskill (University of California Davis, USA) discussed updates on HCC surgery guidelines within HICs. Dr. Jim Littlejohn (University of California Davis, USA) provided a recorded lecture on key aspects of anesthesia care for liver surgery patients, highlighting key considerations that may serve as targets for capacity improvement in Tanzania. Finally, Dr. Hossam Soliman (Menoufia University, Egypt) discussed pushing the boundaries in liver transplant and ablation.

## Liver cancer themed scientific abstract presentations

The scientific sessions of the conference included abstracts on original research and case reports featuring researchers from across Tanzania. This session was opened with a combined presentation by Dr. Katherine VanLoon (University of California San Francisco, USA) and Dr. Beatrice Mushi (MUHAS, Tanzania) presented on building a research program and highlighted the equitable partnership within the Global Cancer Program between UCSF and MUHAS. Dr. Mike Didas (General Surgery Resident, Aga Khan Hospital, Dar es Salaam) presented on ‘Taming the liver Goliath - A case-based exploration of Giant Hepatic Hemangioma’. Dr. Arnold Orchanda (General Surgery Resident, Aga Khan Hospital, Dar es Salaam) presented on ‘A 5-year disease-free survival following right hepatectomy for Hepatitis B-associated HCC’. Dr. Yushan Swai (General Practitioner, Ampola Tasakhtaa Hospital, Zanzibar) presented on the ‘Incidence of HCC and its associated factors at MNH’. Dr. Irene Nguma (Oncology, Mbeya Zonal Referral Hospital, Mbeya) presented on HCC in the Southern Highlands of Tanzania. Dr. Balowa Musa Baraka (IR, MNH, Dar es Salaam) presented on the diagnostic accuracy of HCC through imaging in Tanzania. Dr. Kennedy Hisso (General Surgery, Kilimanjaro Christian Medical Center, Kilimanjaro) presented on the HCC experience in the Northern Zone of Tanzania.

## Establishing national guidelines for HCC in Tanzania

A pinnacle feature of the TLCC 2025 was to leverage the congregation of experts to collaborate on consensus statements for the treatment of HCC in Tanzania. Preconference activities included surveying participants on agreement with key diagnostic, treatment and capacity prioritisation statements. Dr. Jerry Ndumbalo (Clinical and Radiation Oncology, Tanzania) introduced the session, discussing international guidelines into the context of Tanzania. He presented on the prior efforts made with, the Africa Guidelines for HCC Build-Up Process of 2021, the National Comprehensive Cancer Network Harmonised Guidelines for SSA, the Tanzania National Cancer Treatment Guidelines of 2020, and the Standard Treatment Guidelines and National Essential Medicines List for Tanzania of 2021. This was the perfect segway for Dr. Cameron Gaskill (Surgical Oncology, USA) to begin leading the group through a Delphi process of establishing new national guidelines for HCC management in Tanzania passed on preconference survey results. Consensus statements were established if over 90% were in agreement. Each statement not in agreement was discussed and modified in multiple rounds, either reaching agreement or decidedly not reaching agreement. This was done with the vision of revising these recommendations every year based on the changes made to liver cancer capacity within the country.

## Closing remarks

Dr. Ally Mwanga closed the conference with words of advice for the attendees on how to move forward and unite as a community in the fight against liver cancer in Tanzania. Dr. Mwanga commended the attendees for participating in the conference and expressing their commitment to the field. He expressed his sincere gratitude to the zonal delegates that participated from all over Tanzania. He noted that more work needs to be done on the national level and expressed his hopes for a unified national MLTB. He also announced the creation of the Tanzanian Hepato-Pancreato-Biliary Association (THPBA) as an expansion of the TLCG.

## Conference impact on attendees

This conference received positive feedback from attendees with 100% of survey respondents reporting being either ‘very satisfied’ or ‘satisfied’ with the event, 100% would recommend it to a colleague and 100% indicating they would attend next year’s conference. Furthermore, the quality of presentations was highly regarded, with 95% of attendees rating them as 4 or 5 out of 5. The organisation and logistics of the event also received strong approval, with 96% of respondents rating them as 4 or 5 out of 5. All attendees (100%) reported that the conference either met or exceeded their expectations. Finally, a significant 99% of respondents indicated they are ‘very likely’ or ‘likely’ to attend another TLCG event in the future.

## Conclusion

The second TLCC accomplished its goal of bringing together healthcare practitioners from across Tanzania and SSA dedicated to the prevention, diagnosis and treatment of liver cancer. This conference took a unique and powerful approach aimed at: 1) building awareness among local and international healthcare providers on the status of liver cancer management in Tanzania 2) promote a multidisciplinary approach to the clinical care of liver cancer patients in Tanzania and 3) foster local and international collaboration on liver cancer research. While this event has come to an end, it only marks the beginning of a new chapter as local stakeholders look forward to the creation of HCC guidelines for Tanzania, the national MLTB and the creation of the THPBA. As Tanzania’s second conference on the subject, the TLCC2025 demonstrated the continued forward momentum in the fight against liver cancer.

## Conflicts of interest

The authors declare that they have no conflicts of interests.

## Funding

This research did not receive any specific grant from funding agencies in the public, commercial or not-for-profit sectors.
